# Stressful Family Contexts and Health in Divorced and Married Mothers

**DOI:** 10.1093/geroni/igab046.1526

**Published:** 2021-12-17

**Authors:** Eric Klopack, Kandauda Wickrama

**Affiliations:** 1 University of Southern California, Los Angeles, California, United States; 2 University of Georgia, Athens, Georgia, United States

## Abstract

Using prospective data over 25 years from a sample of 416 women, the first objective of the current study was to investigate the biopsychosocial process over the second-half of the life course comparing mothers with diferent marital histories. The second objective was to investigate this biopsychosocial process for 296 maried mothers focusing on their marital quality over middle years. The results suggested that, compared to being married, divorcing in early-midlife launched an adverse biopsychosocial process for women leading to physical pain, physical limitations, and depressive symptoms over their mid-later years, largely through early-midlife financial stress, regardless of later recoupling. However, subsequent financial stress did not influence divorced mothers’ later-life health problems, suggesting their development of resilience. For consistently married mothers, both marital stress and financial stress uniquely influenced all three health problems throughout their mid-later years. For all mothers, these health problems progressed over mid-later years, as indicated through their stabilities and mutual influences, and these health problems also selected mothers into further escalating financial and marital stress over their mid-later years. Elucidating differential short- and long-term health influences of marital and financial stressors for divorced and married mothers provides a potentially useful information for targeted early preventive intervention efforts and policy formation. Such interventions can promote and develop resiliency factors, thereby aiding middle-aged mothers to prevent from adverse biopsychosocial processes.

